# Evaluation of four molecular methods to detect *Leishmania* infection in dogs

**DOI:** 10.1186/s13071-017-2002-2

**Published:** 2017-03-13

**Authors:** Andreia Albuquerque, Lenea Campino, Luís Cardoso, Sofia Cortes

**Affiliations:** 1 Global Health and Tropical Medicine, GHTM, Instituto de Higiene e Medicina Tropical, IHMT, Universidade Nova de Lisboa, UNL, Rua da Junqueira 100, 1349-008 Lisbon, Portugal; 2 Present address: Institut für Zelluläre Chemie, Medizinische Hochschule Hannover, Carl-Neuberg-Str. 1, 30625 Hannover, Germany; 3 Department of Biomedical Sciences and Medicine, Campus Gambelas, Universidade de Faro, Faro, Portugal; 4 Department of Veterinary Sciences, School of Agrarian and Veterinary Sciences, University of Trás-os-Montes e Alto Douro, UTAD, Quinta de Prados, 5000-801 Vila Real, Portugal

**Keywords:** Dogs, *Leishmania*, Canine leishmaniasis, Subclinical infection, Molecular diagnosis, Nested SSU rRNA-PCR

## Abstract

**Background:**

Canine leishmaniasis, a zoonotic disease caused by *Leishmania infantum* vectored by phlebotomine sand flies, is considered a relevant veterinary and public health problem in various countries, namely in the Mediterranean basin and Brazil, where dogs are considered the main reservoir hosts. Not only diseased dogs but also those subclinically infected play a relevant role in the transmission of *L. infantum* to vectors; therefore, early diagnosis is essential, under both a clinical and an epidemiological perspective. Molecular tools can be a more accurate and sensitive approach for diagnosis, with a wide range of protocols currently in use. The aim of the present report was to compare four PCR based protocols for the diagnosis of canine *Leishmania* infection in a cohort of dogs from the Douro region, Portugal.

**Results:**

A total of 229 bone marrow samples were collected from dogs living in the Douro region, an endemic region for leishmaniasis. Four PCR protocols were evaluated for *Leishmania* DNA detection in canine samples, three single (ITS1-PCR, MC-PCR and Uni21/Lmj4-PCR) and one nested (nested SSU rRNA-PCR). Two of the protocols were based on nuclear targets and the other two on kinetoplastid targets. The higher overall percentage of infected dogs was detected with the nested SSU rRNA-PCR (37.6%), which also was able to detect *Leishmania* DNA in a higher number of samples from apparently healthy dogs (25.3%). The ITS1-PCR presented the lowest level of *Leishmania* detection.

**Conclusions:**

Nested SSU rRNA-PCR is an appropriate method to detect *Leishmania* infection in dogs. Accurate and early diagnosis in clinically suspect as well as apparently healthy dogs is essential, in order to treat and protect animals and public health and contribute to the control and awareness of the disease.

## Introduction

Canine leishmaniasis (CanL), caused by the protozoan parasite *Leishmania infantum*, is a veterinary medical and public health problem in different Mediterranean countries, namely those of southern Europe, and also in Brazil, in which dogs are considered the primary domestic reservoir host for the human infection. According to Moreno & Alvar [1], it is estimated that at least 2.5 million dogs are infected in south-western Europe. In addition, there is an evident northward expansion of CanL in Europe, as demonstrated by epidemiological studies in countries from the eastern part of the continent [2].

Currently, laboratorial diagnosis of CanL is usually performed by direct parasitological examination and/or serological methods, which are time consuming and may lack accuracy. Therefore, more sensitive and specific methods, namely molecular diagnostic tools, are essential to detect *Leishmania* infection, both in clinically suspected and apparently healthy dogs, since the latter group can also be a source of the parasite to the phlebotomine vectors [3]. According to a recent meta-analysis on CanL carried out in Iran, most infected dogs presented no clinical signs [4].

The polymerase chain reaction (PCR) is nowadays a simple and valid molecular tool to detect *Leishmania* spp. in different clinical samples, as well as to identify the parasite species, strains and genotypes [5]. There are currently a large number of protocols with sensitivities and specificities that depend on different factors such as the DNA extraction method, clinical material, primers, target copy numbers, and technical conditions [6]. With PCR, diagnosis of CanL has shown a considerable improvement, with sensitivities of 90–100% in clinically suspected or parasitological confirmed cases (reviewed in [6]). However, when dealing with early diagnosis and the detection of parasite in subclinical cases, which can reach 80% of dogs [7], sensitivity might be much lower.

In this study, we compared four PCR based protocols using different DNA targets (small sub-unit rRNA gene, ITS-1 and kDNA) with Novy-MacNeal-Nicolle (NNN) culture in a cohort of dogs from a region of Portugal where leishmaniasis is endemic. All the protocols used were previously established in IHMT for the diagnosis of human leishmaniasis. However, for the diagnosis of CanL only MC-PCR has been used, with no comparison of performances made before. Under these circumstances, we aimed at selecting an appropriate method for the detection of *Leishmania* canine infections.

## Methods

Between July 2011 and October 2012, 229 bone marrow samples were collected from dogs housed in two Animal Municipal Centres in the Douro region, a geographical area where CanL is endemic in Portugal. After physical examination and depending on the presence of clinical manifestations compatible with the disease, such as weight loss, alopecia, lymphadenomegaly, lethargy, pale mucosae and skin lesions [8], the animals were characterized as clinically suspected, i.e. presenting clinical signs (*n* = 150) or apparently healthy, i.e. with no clinical signs (*n* = 79). Bone marrow samples were obtained from the osteochondral joint of the sixth to the ninth ribs, and placed into EDTA tubes and further divided into two parts: one for NNN culture, as described by Maia & Campino [9], and the other one for DNA extraction using a commercial kit (PCR Template Preparation Kit, Roche, Germany).

The presence of *Leishmania* DNA was evaluated by four PCR protocols using different DNA targets, as described in Table 1: (i) nested PCR using the variable part of the small sub-unit rRNA gene (nested SSU rRNA-PCR); (ii) ribosomal internal transcribed spacer 1 (ITS1-PCR); (iii and iv) kinetoplastid minicircle sequences (MC-PCR; Uni21/Lmj4-PCR). In order to assure DNA integrity, a PCR using the canine β-actin gene was performed using 2 μl of DNA, 10 pmol of each primer (5'-TGA CGG GGT CAC CCA CAC TGT GCC CAT CTA-3' and 5'-CTA GAA GCA TTG CGG TGG ACG ATG GAG GG-3'), 1U de *Taq* polymerase and buffer 1× (Promega, Madison, WI, USA), 1.5 mM MgCl_2_, 0.2 mM dNTPs (Bioline, London, UK), under the following conditions: 40 cycles each denaturation at 94 °C (30 s), annealing at 64 °C (30 s) and extension at 72 °C (30 s). The amplified products presented a fragment of 283 bp.

**Table 1:** PCR protocols used for *Leishmania* detection in dog samples

Protocol	Primer sequence	Amplicon size (bp)	PCR conditions (final concentration)	Cycling conditions	Reference
Nested SSU rRNA-PCR	1^st^ PCR: R221: GGTTCCTTTCCTGATTTACG R332: GGCCGGTAAAGGCCGAATAG	603	10 μl DNA, 2 mM MgCl_2_, 0.2 mM dNTPs, 15 pmol primers, 1.4U *Taq*, 1× buffer (Promega)	den.: 94 °C (30'); ann.: 60 °C (30'); ext.: 72 °C (30'); 35 cycles	[34]
	2^nd^ PCR: R223: TCCATCGCAACCTCGGTT R333: AAAGCGGGCGCGGTGCTG	358	5 μl 1^st^ PCR prod^a^., 2 mM MgCl_2_, 0.2 mM dNTPs, 1.5 pmol primers, 0.7U *Taq*, 1× buffer (Promega)	den.: 94 °C (30'); ann.: 65 °C (30'); ext.: 72 °C (30'); 32 cycles	[10]
ITS1-PCR	LITSR: CTGGATCATTTTCCGATG L5.8S: TGATACCACTTATCGCACTT	311	2 μl DNA, 1.5 mM MgCl_2_, 0.2 mM dNTPs, 25 pmol primers, 1U *Taq*, 1× buffer (Promega)	den.: 95 °C (20'); ann.: 53 °C (20'); ext.: 72 °C (60'); 32 cycles	[35]
MC-PCR	MC1: GTTAGCCGATGGTGGTCTTG MC2: CACCCATTTTTCCGATTTTG	447	2 μl DNA, 3 mM MgCl_2_, 0.2 mM dNTPs, 15 pmol primers, 1U *Taq*, 1× buffer (Promega)	den.: 94 °C (30'), ann.: 60 °C (30'); ext.: 72 °C (20'); 30 cycles	[13]
Uni21/Lmj4-PCR	Uni21: GGGGTTGGTGTAAAATAGGCC Lmj4^b^: CTAGTTTCCCGCCTCCGAG	800	2 μl DNA, 1.5 mM MgCl_2_, 25 pmol primers, 12,5 μl Biomix (Bioline)	den.: 94 °C (30), ann.: 62 °C (30'); ext.: 72 °C (45'); 35 cycles	[36]

*Abbreviations*: *bp* base pairs, *den.* denaturation, *ann.* annealing, *ext.* extension, *prod.* product, *PCR* polymerase chain reaction, *MC* minicircle, *ITS1* internal transcribed spacer 1, *rRNA* ribosomal RNA gene, *SSU* small subunit

^a^PCR product was previously diluted 1:200 in ultra-pure water

^b^Uni21 primer based on a conserved region of a *Leishmania major* kinetoplastid minicircle sequence, and Lmj4 based on the variable region of the same *L. major* sequence

The *Z*-test for absolute difference between two proportions was used to compare proportions by means of the StatLib free software, with a probability *P*-value < 0.05 being considered as statistically significant.

## Results and discussion

A significant percentage of positive samples was found with the nested SSU rRNA-PCR protocol (37.6%, *P* < 0.001; Table 2). Moreover, with this protocol we detected a higher percentage of positive samples in apparently healthy dogs (25.3%) than with the other tested protocols, a fact that strongly suggests it to be a more adequate tool for detection of *Leishmania*, especially in subclinically infected dogs. These molecular markers are able to identify *Leishmania* parasites at the genus level. This same protocol was previously shown to have high sensitivity and to be a useful tool for human leishmaniasis diagnosis, monitoring the success of treatment, and predicting relapses in patients with HIV infection [10]. Nevertheless, this methodology, which involves a second PCR step, may be prone to contamination among samples, thus requiring higher attention in performing dilution of the first amplicons and in the second PCR step. The analysis of all positive samples along with intercalated known negative samples was repeated in order to exclude potential contaminations.

**Table 2:** Positive samples, analysed by the different PCR protocols, from 150 dogs clinically suspected of leishmaniasis and from 79 apparently healthy dogs

PCR protocol	No. of positive samples (%)		
	CS dogs (*n* = 150)	AH dogs (*n* = 79)	All dogs (*n* = 229)
Nested SSU rRNA-PCR	66 (44.0)^a^	20 (25.3)^a^	86 (37.6)^d,e,f^
ITS1-PCR	18 (12.0)^b^	2 (1.3)^b^	20 (8.7)^d^
MC-PCR	28 (18.7)^c^	5 (6.3)^c^	33 (14.4)^e^
Uni21/Lmj4-PCR	23 (15.3)	10 (12.7)	33 (14.4)^f^

*Abbreviations*: *AH* apparently healthy, *CS* clinically suspected, *ITS1* internal transcribed spacer 1, *MC* minicircle, *PCR* polymerase chain reaction, *rRNA* ribosomal RNA gene, *SSU* small subunit, *Uni21/Lmj4* Uni21 primer based on a conserved region of a *Leishmania major* kinetoplastid minicircle sequence, and Lmj4 based on the variable region of the same *L. major* sequence

Only statistically significant differences are shown: ^a^ *Z* = 2.76, *P* = 0.006; ^b^ *Z* = 2.41, *P* = 0.016; ^c^ *Z* = 2.53, *P* = 0.012; ^d^ *Z* = 7.31, *P* < 0.001; ^e,f^ *Z* = 5.65, *P* < 0.001

ITS1-PCR presented the lowest number of positive samples (8.7%; Table 2). As for the nested SSU rRNA-PCR protocol, this one also detects *Leishmania* at the genus level. This limitation regarding the non-distinction at the species level can be critical in regions where more than one *Leishmania* spp. is present. When using the ITS1 marker, an additional RFLP analysis should be performed for species identification [5]. However, it may not be the most appropriate method, as it does not differentiate within the *L. donovani* complex [11]. Therefore, sequencing should be used as complementary to this analysis [12].

Kinetoplastid MC-PCR protocol was able to detect and identify *Leishmania* at the complex level. This protocol, with primers targeted to a kinetoplastid minicircle sequence of the *Leishmania donovani* complex [13], allowed the identification of 33 positive samples (14.4%; Table 2), mainly in clinically suspected dogs (18.7%, *P* = 0.012; Table 2).

In Uni21/Lmj4-PCR protocol, the primer pair Uni21/Lmj4 was developed based on a *Leishmania major* minicircle kinetoplastid sequence. This protocol, based on species-specific differences in amplicons size, differentiate Old World *Leishmania* species, namely *L. infantum.* Although with the same overall percentage of positive samples as the MC-PCR, this method allowed the detection of *Leishmania* DNA in a higher number of apparently healthy dogs.

The NNN culture detected only three positive bone marrow samples. Out of these, IMT 401 (MCAN/PT/13/IMT401) was sent to the Centre National de Référence des Leishmanioses (Montpellier, France) and typed by multilocus enzyme electrophoresis (MLEE) [14] as *L. infantum* MON-1, the most common zymodeme in both humans and dogs in the Mediterranean basin [15]. The low sensitivity of cultural parasitological techniques has also been described by other authors [16, 17]. Moreover, cultures are prone to contamination and may take up to 4 weeks to provide a definitive diagnosis. These limitations reinforce the need for more sensitive techniques for the diagnosis of this disease, as is the case with molecular diagnostic PCR techniques.

Simple PCR methods targeting kDNA minicircles have been described as having higher sensitivity than SSU rRNA-PCR in human samples. However, the improvement of the latter, by using a nested approach, may increase its sensitivity, further allowing full species identification when combined with sequencing analysis [18].

The large number of subclinical *Leishmania* infections in dogs has been well proven in epidemiological studies worldwide, mainly by using serological methods but also with advanced molecular methods [4, 19]. In the Douro region, Cardoso et al. [20] highlighted this high percentage of subclinical infections and found that out of 60 seropositive animals, 51 (85%) had no clinical signs compatible with CanL. Similar results were found in other geographic areas of the Mediterranean basin, particularly in France, Italy, Spain, central and southern Portugal, Turkey, and also in Brazil [21–26].

The high proportion of no patent disease may be related to the development of some level of cellular immune response, characterized by the production of Th1 cytokines, such as IFN-γ, IL-2 and TNF-α, which is known to limit the progression of the infection [27, 28]. Additionally, these apparently healthy dogs can act as reservoirs, leading to the spread of infection with increases in both canine prevalence and human incidence. Thus, reinforcing control measures as well as performing effective clinical management, by using sensitive molecular methods in animals with and without clinical signs, could improve diagnosis of the disease at an early stage [3, 29, 30].

It has already been described that infectiousness increases in dogs with clinical signs, as there is a higher probability of transmission of the parasites to the phlebotomine vectors and spreading of CanL, than in apparently healthy dogs. However, it is imperative to stress that the latter ones may also contribute to parasite transmission [3, 31, 32].

This study showed, from the four tested protocols, that nested SSU rRNA-PCR was the most appropriate to detect *Leishmania* infection in dogs, especially in subclinical cases. Although only 44% of the positive samples were detected by nested SSU rRNA-PCR in bone marrow of clinical suspected dogs, it is important to note that the use of this biological sample for diagnosis of CanL is not a consensus. It has been described that the use of other samples such as peripheral blood, skin or conjunctiva could be more appropriate [16, 22]. On the other hand, it is possible that the threshold limit of the PCR test might be below the detection level as amastigotes tend to disseminate to other organs or even due to “parasite silencing” caused by the host defence mechanisms [33].

## Conclusions

From an epidemiological point of view, for diagnosis of canine *Leishmania* infection, it is of extreme relevance the selection of an appropriate type of biological material as well as an adequate protocol. Furthermore, in order to contribute to the control of leishmaniasis in dogs and humans, actions should be directed to both dogs with and without clinical signs of leishmaniasis and also to increase the awareness of dog owners regarding the implementation of prophylactic measures.
